# A Voltage-Tuned Terahertz Absorber Based on MoS_2_/Graphene Nanoribbon Structure

**DOI:** 10.3390/nano13111716

**Published:** 2023-05-24

**Authors:** Omnia Samy, Mohamed Belmoubarik, Taiichi Otsuji, Amine El Moutaouakil

**Affiliations:** 1 College of Engineering, United Arab University, Al Ain P.O. Box 15551, United Arab Emirates; 2 International Iberian Nanotechnology Laboratory, INL, Av. Mestre José Veiga s/n, 4715-330 Braga, Portugal; 3 Research Institute of Electrical Communication, Tohoku University, 2-1-1 Katahira, Aoba-ku, Sendai 980-8577, Japan

**Keywords:** THz absorbers, graphene, monolayer MoS_2_, monolayer black phosphorous, THz applications, nanoribbons

## Abstract

Terahertz frequency has promising applications in communication, security scanning, medical imaging, and industry. THz absorbers are one of the required components for future THz applications. However, nowadays, obtaining a high absorption, simple structure, and ultrathin absorber is a challenge. In this work, we present a thin THz absorber that can be easily tuned through the whole THz range (0.1–10 THz) by applying a low gate voltage (<1 V). The structure is based on cheap and abundant materials (MoS_2_/graphene). Nanoribbons of MoS_2_/graphene heterostructure are laid over a SiO_2_ substrate with an applied vertical gate voltage. The computational model shows that we can achieve an absorptance of approximately 50% of the incident light. The absorptance frequency can be tuned through varying the structure and the substrate dimensions, where the nanoribbon width can be varied approximately from 90 nm to 300 nm, while still covering the whole THz range. The structure performance is not affected by high temperatures (500 K and above), so it is thermally stable. The proposed structure represents a low-voltage, easily tunable, low-cost, and small-size THz absorber that can be used in imaging and detection. It is an alternative to expensive THz metamaterial-based absorbers.

## 1. Introduction

THz frequency is the range of frequencies lying between 300 GHz and 10 THz that are characterized by their low energy and high penetration. The characteristics of these frequencies have enabled them to be preferable in medical applications, imaging, security scanning, the food industry, and communication [1,2,3]. THz frequencies are promising in biomedical detection (cancer diagnosis), imaging, and sensing, since they represent a label-free, nondestructive detecting technique [4]. From an economic perspective, the global market investment in THz applications is rapidly increasing and is expected to hit USD 2.87 billion by 2030 at a growth rate of about 23.8% [5]. Research is directed towards developing THz emitters, receivers, and detectors with good performance and quality. Studies are concerned with using cheap and abundant materials such as graphene, instead of III-V semiconductors that require expensive and complicated fabrication techniques [6,7].

Graphene, the 2D allotrope of carbon, has a monolayer thickness of approximately 0.34 nm. It is a zero-band-gap semiconductor that has high electron mobility (~200,000 cm^2^/Vs) and high optical transparency (97.4%) [8,9]. Graphene has plasma oscillations in the THz frequency range that can be modulated through applying an electric field [10,11]. It has absorption in the THz region [10] and it is a broadband transmitter at higher frequencies [12,13,14]. When graphene is excited with perpendicular electromagnetic waves, the surface plasmons interact with the incident waves producing surface plasmon polaritons (SPPs). This leads to a transverse TM (TE) electromagnetic mode that does not exist in conventional 2D systems with parabolic electron dispersion. Despite these favourable electronic and optical characteristics, the zero bandgap of graphene hinders its applications. Possible ways to increase the bandgap of graphene are stripping it into nanoribbons or developing heterostructures. MoS_2_/graphene heterostructure has desirable electronic properties and a high on/off ratio (~10^8^) that open the way for different electronic applications [15,16].

MoS_2_ is a transition metal dichalcogenide (TMD) with a bulk indirect bandgap of 1.2 eV and a monolayer direct bandgap of 1.89 eV. This specific bandgap endows MoS_2_ with unique photoluminescence characteristics where it can be used as a biomarker for cancer and DNA detection [17,18,19,20,21]. Since MoS_2_’s bandgap is larger than the energy of the THz spectrum, no photocarriers are generated when it is excited with THz frequencies [22]; however, it can have very small absorption peaks due to intraband transitions [23]. A MoS_2_/graphene heterostructure has more desirable properties than the monostructures of graphene or MoS_2_. The heterostructure has a larger bandgap than graphene and an enhanced carrier mobility compared to MoS_2_ [15,24]. The high transparency of MoS_2_ in the THz region and the Fermi level tunability of graphene enable MoS_2_/graphene heterostructures to have promising electronic and optoelectronic applications [25,26,27]. The absorption behaviour of the heterostructure resembles that of graphene in the THz range. The vertical bias has nearly no influence on MoS_2_’s optical properties at low energy frequencies [28,29], but it enhances the charge transfer between MoS_2_ and graphene.

Due to the favourable characteristics of graphene and MoS_2_, a lot of work has been carried out to investigate the behaviour of this heterostructure. The MoS_2_/graphene heterostructure has better absorptance than graphene [30]. Applying an electric field to graphene only or the MoS_2_/graphene heterostructure affects its bandgap and its optical properties [27,31]. Vertical electromagnetic waves excite the surface plasmons in graphene nanoribbons which are in the THz range, as described by [11]. Local surface plasmons are also excited in MoS_2_ under electromagnetic excitation. These surface plasmons play an important role in wave absorption. Moreover, the charge transfer through the heterojunction MoS_2_/graphene enhances the generation recombination process, resulting in better absorption. MoS_2_/graphene heterostructures are favourable in field effect transistors (FETs) in electronics. In [32], a FET sensor based on the MoS_2_/graphene heterostructure was proposed where the charge transfer through the heterojunction results in better chemical sensing and the FET has better stability in dry air. It also has higher sensitivity and better signal-to-noise ratio (SNR). A new transistor based on MoS_2_/graphene in [33] showed a steep SS of 37.9 mV/dec at ambient temperature, which can be enhanced by modifying the transistor structure to a gate all around (GAA) FET. The FET has a current on/off ratio of $10^{8}$, which opens the way for low-power and high-performance electronics applications that can substitute silicon-based FETs. Graphene can reduce the contact resistivity in MoS_2_ FETs. A graphene/MoS_2_ heterojunction FET with a 30 nm channel length has no short channel effects and a has drain-induced barrier lowering of 0.92 *V*/*V*. When graphene electrodes were doped with nitrogen, an increase in the device current by 214% was detected and the field effect mobility was four times that of undoped graphene electrodes [34]. The absorption of graphene and MoS_2_ was previously studied, where low THz absorption peaks were detected in graphene–MoS_2_ microribbon grating [30]. The absorption frequency depends on the microribbon width and the grating spacing. The absorption of MoS_2_ with other materials such as black phosphorous was investigated in [23], where a MoS_2_/BP structure had six absorption bands in the THz region.

None of the previous studies discussed the absorptance of the heterostructure in the THz region under the effect of direct voltage application. Tunable THz absorbers can also be developed using metamaterials [35]; however, here, we are concerned with using low-cost and abundant materials. In this work, we investigate the absorption of infinite MoS_2_/graphene nanoribbons under vertical gate bias. The heterostructure has enhanced absorptance in the whole THz region. The frequency of absorptance is easily tuned by varying the applied voltage. It is a cheap alternative for metamaterial THz absorbers. It can be utilized in many applications such as the sensing and detection of THz radiations [36].

## 2. Materials and Methods

**Figure 1:**
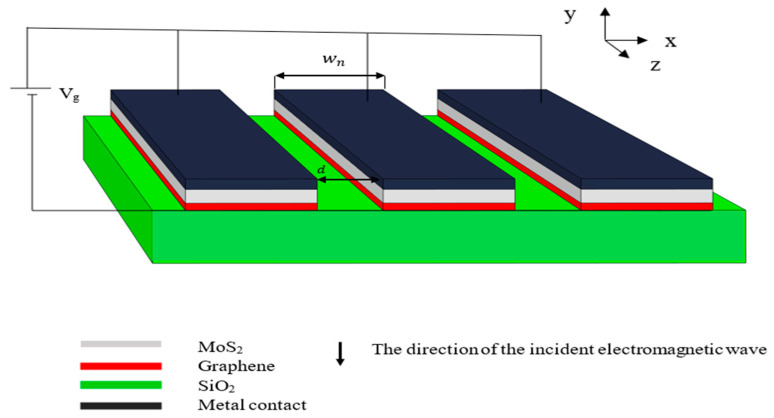
The schematic of the infinite nanoribbon structure with a SiO_2_ substrate and nanoribbons of graphene and MoS_2_ with applied gate voltage, V_g_, and incident THz electromagnetic wave.

The proposed nanoribbon structure is based on the schematic in Figure 1. Infinite nanoribbons of monolayer graphene with 0.345 nm thickness were laid over a SiO_2_ substrate with 5 nm thickness. A monolayer of MoS_2_ with 0.65 nm thickness stripped into nanoribbons was laid over the graphene nanoribbons to have an infinite array of MoS_2_/graphene nanoribbons in the horizontal direction. The nanoribbon width, $w_{n}$, was 90 nm and the spacing, d, between the nanoribbons was 10 nm. A vertical electromagnetic field was applied in the vertical direction to excite the SPPs [30]. The incident wave had an electric field in the x-direction and a gate voltage, V_g_, was applied to the MoS_2_ nanoribbons through Au metal contacts which have good compatibility with graphene and MoS_2_ [37,38]_._ The metal contacts only served as a means of voltage application. They did not have any effect on the characteristics of the design. Since they were very thin (less than 1 nm), they transmitted all the incident light and did not reflect any. A thin layer of h-BN was then laid over the structure. The calculations were based on the finite element method (FEM) used by COMSOL Multiphysics software. The structure was built and simulated using 2D simulation with applying periodic conditions on the vertical sides to solve for an infinite periodic array structure. The wave optics module, electromagnetic wave frequency domain (EWFD), was used.

The unique band structure of graphene leads to the excitation of specific surface plasmons when they are subjected to electromagnetic waves, as compared to conventional 2D electron systems [39,40]. To ensure the formation of the SPPs and the propagation of TM (TE) modes along graphene nanoribbons, the imaginary part of optical conductivity must exist, whether positive for TM or negative for TE. The best way to solve for the conductivity of graphene is using the Kubo formalism, which accounts for both interband and intraband transitions. The optical conductivity of graphene is represented by Equations (1)–(3) [41,42].

(1) $$\sigma_{\mathrm{intra}}\left(\omega \right)=i\frac{q^{2}}{\pi \hbar^{2}\left(\omega +{i\tau }^{-1}\right)}\times \left(\mu_{c}+2K_{B}T\times \ln\left(e^{-\frac{\mu_{c}}{K_{B}T}}+1\;\right)\right)$$

(2) $$\sigma_{\mathrm{inter}}\left(\omega \right)=i\frac{q^{2}}{4\pi \hbar }\ln\frac{2\left|\mu_{c}\right|-\hbar \left(\omega +{i\tau }^{-1}\right)}{2\left|\mu_{c}\right|+\hbar \left(\omega +{i\tau }^{-1}\right)}$$

(3) $$\sigma_{\mathrm{total}}\left(\omega \right)=\sigma_{\mathrm{intra}}\left(\omega \right)+\sigma_{\mathrm{inter}}\left(\omega \right)$$

where q is the electronic charge, ℏ  is the reduced Planck’s constant (h/2π), ω is the angular frequency, ${\;K}_{B}$ is the Boltzmann constant, T is the temperature, $\mu_{c}$ is the chemical potential, and τ is the momentum relaxation time, defined as

(4) $$\mu_{c}=\hbar v_{f}\sqrt{{\pi n}_{g}}\;$$

(5) $$\tau =\frac{\mu_{c}m_{u}}{{q\;v}_{f}^{2}}$$

and $v_{f}$ is the Fermi velocity, taken as $10^{6}$ m/s; $m_{u}$ is the impurity-limited direct current mobility, taken as $1\;m^{2}/V$; and $n_{g}$ is the carrier concentration of graphene and is defined by

(6) $$n_{g}=\frac{V_{g}\epsilon_{o}\epsilon_{r}}{{q\;d}_{\mathrm{sub}}}$$

where $\epsilon_{o}\epsilon_{r}$ is the vacuum and relative substrate permittivities, respectively, and $d_{\mathrm{sub}}$ is the substrate thickness. In our case, we can adopt a capacitor model for the charge concentration of graphene by

(7) $$n_{g}=\frac{V_{g}\epsilon_{o}}{q\;\left(\frac{d_{{\mathrm{MoS}}_{2}}}{\epsilon_{{\mathrm{MoS}}_{2}}}+\frac{d_{\mathrm{Gr}}}{\epsilon_{\mathrm{Gr}}}\right)}$$

where $d_{{\mathrm{MoS}}_{2}},{\;d}_{\mathrm{Gr}}$ are the MoS_2_ and graphene thickness, taken as 0.65 nm and 0.34 nm, respectively; $\epsilon_{{\mathrm{MoS}}_{2}},\;\epsilon_{\mathrm{Gr}}$ are the static relative permittivities of MoS_2_ and graphene, taken as 3.7 and 5.6, respectively. Since monolayer MoS_2_ has a large bandgap with respect to THz waves, its optical conductivity is represented by the Drude model in Equation (8), where only intraband transitions dominate.

(8) $$\sigma_{{\mathrm{MoS}}_{2}}=\frac{{\mathrm{iq}}^{2}n_{{\mathrm{MoS}}_{2}}}{m_{{\mathrm{MoS}}_{2}}^{*}\left(\omega +{i\tau }^{-1}\right)}$$

where $n_{{\mathrm{MoS}}_{2}}=1.2\times 10^{12}{\;\mathrm{cm}}^{-2}$ is the carrier concentration of undoped monolayer MoS_2_ and $m_{{\mathrm{MoS}}_{2}}^{*}=0.53m_{e}$ is the effective mass, τ = 0.17 × $10^{-12}$ s is the carrier relaxation time [43,44]. The complex permittivity of MoS_2_ ($\epsilon_{{\mathrm{MoS}}_{2}}^{\prime })$ is then calculated from Equation (9)

(9) $$\epsilon_{{\mathrm{MoS}}_{2}}^{\prime }=\epsilon_{{\mathrm{MoS}}_{2}}+\frac{{i\sigma }_{{\mathrm{MoS}}_{2}}}{\epsilon_{0}{\omega d}_{{\mathrm{MoS}}_{2}}}=\epsilon_{r}+i\epsilon_{i}$$

where $\epsilon_{r}$ and $\epsilon_{i}$ are the real and imaginary parts of MoS_2_ permittivity, the real part of the refractive index is $n=\sqrt{\frac{\epsilon_{r}}{2}+\frac{1}{2}\sqrt{\epsilon_{r}^{2}+\epsilon_{i}^{2}}}$, and the extinction coefficient is $\kappa =\frac{\epsilon_{i}}{2n}$. In the case of Drude model representation, we only have a positive imaginary conductivity term that accounts for a TM mode. The complex permittivities of Au and SiO_2_ in the THz region were calculated from [45,46], respectively. Since there is little variation in the real and imaginary parts of the refractive index of SiO_2_ and Au in the THz range, we used average constant values of 2.15 for SiO_2_ and 160 + i600 for Au.

**Figure 2:**
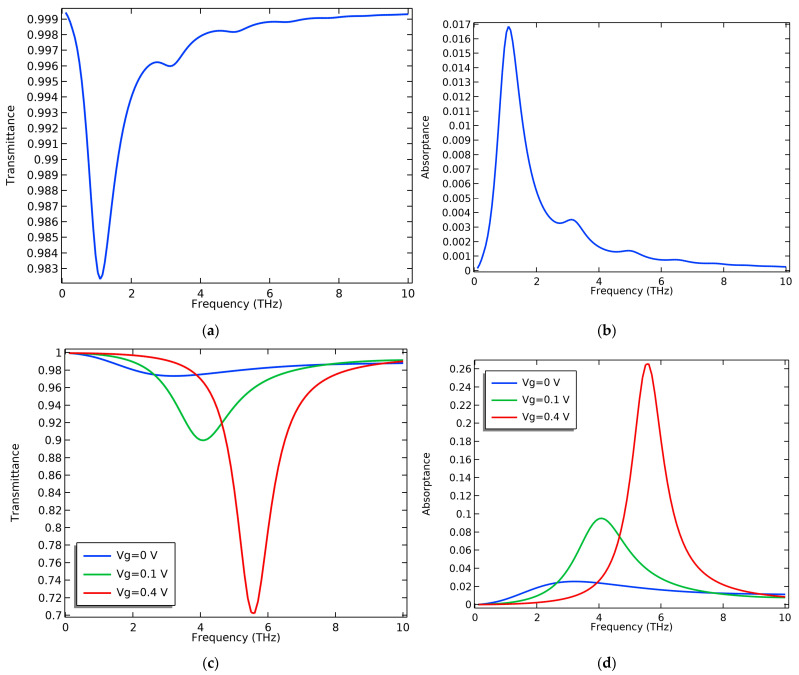
The transmittance and absorptance of MoS_2_ monolayer nanoribbon over SiO_2_ and graphene nanoribbon over SiO_2_: (**a**) The transmittance of MoS_2_; (**b**) The absorptance of MoS_2_; (**c**) The transmittance of graphene; (**d**) The absorptance of graphene. d_sub_ = 5 nm.

MoS_2_ has high transmission in the THz region, and it has a plasmon frequency outside the THz region at approximately 16.77 THz [47]. The transmission of a monolayer MoS_2_ nanoribbon over SiO_2_ is plotted in Figure 2a which agrees with the values in [47] for monolayer MoS_2_. Equations (3) and (8) were used to plot the absorption and transmittance of graphene-only nanoribbons and MoS_2_-only nanoribbons over the SiO_2_ substrate, as illustrated in Figure 2. The absorption peaks of graphene and MoS_2_ in Figure 2b,d can be interpreted due to the excitation of surface plasmons with the same propagation mode as the incident wave. In the case of MoS_2_, local surface plasmons are excited and a very small absorption peak exists at a low THz frequency, which is due to the low carrier concentration of MoS_2_. In graphene, the strong excitation of surface plasmons occurs, resulting in higher peaks attributed to its higher carrier concentration. The absorption peak is tuned with voltage due to graphene’s optical conductivity tunability through varying its chemical potential.

## 3. Results and Discussion

### 3.1. The Effect of Vertical Bias

Graphene absorption can be tuned using vertical bias, but MoS_2_ is slightly affected. The gate bias had no effect on MoS_2_ bilayer absorption, and in the monolayer, an increase in absorption was reported at 1.85 eV which was far from the THz region (gate bias applied from 50 to 50 V) [48]. That is why applying a gate voltage on MoS_2_ in the THz range does not affect its performance. Monolayer MoS_2_ can have absorption peaks at the THz region when doped as n-type or p-type (high carrier concentration) and applying a spin–orbit coupling effect [49]; however, we here are concerned with intrinsic MoS_2_ and the simple application of a gate voltage. The refractive index of MoS_2_ was found to be independent on the vertical gate bias, which is attributed to its large bandgap compared to the THz range and its low carrier concentration [28].

**Figure 3:**
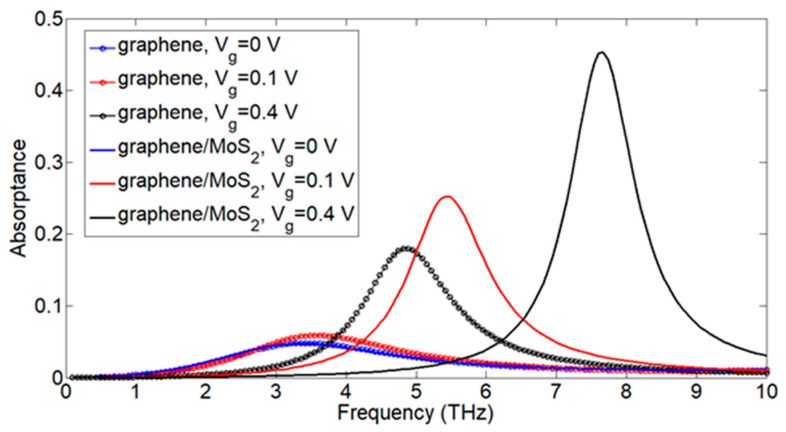
The nanoribbon structure absorptance in case of graphene only (marked lines) and MoS_2_/graphene heterostructure (solid lines) at different gate voltages Vg = 0, 0.1, 0.4 V.

In the case of the MoS_2_/graphene heterostructure, the bandgap increased, forming mini bandgaps [24,50] with a possible charge transfer between MoS_2_ and graphene. When applying a positive vertical gate bias on MoS_2_, holes were encouraged to migrate from the MoS_2_ to the graphene, which means a wider bandgap for the MoS_2_/graphene heterostructure and a higher absorptance frequency (the absorptance frequency was shifted to the right) as in Figure 3. The absorption of the graphene-only nanoribbons is compared to that of the heterostructure at different gate voltages in Figure 3. The graphene nanoribbons (marked lines) showed low absorption peaks that slightly moved to the right when increasing the applied voltage. For the MoS_2_/graphene nanoribbons, there was a remarkable increase in the absorption peak that can be finely tuned through the whole THz region by applying voltage. The absorption peak at V_g_ = 0 is nearly the same for graphene-only and the MoS_2_/graphene heterostructure but it obviously increased with higher gate voltages, indicating that the charge transfer between MoS_2_ and graphene can be neglected at zero bias, as suggested by [50]. The enhancement in absorption can also be attributed to the surface plasmon excitation in graphene and MoS_2_. Graphene can have TE and TM modes, while MoS_2_ has only TM modes. The unity between the TM surface plasmon modes of both graphene and MoS_2_ (the imaginary terms of conductivity of graphene and MoS_2_ are positive) results in frequency resonance. When this resonance frequency matches the frequency of the incident light, an increase in absorption frequency is detected.

**Figure 4:**
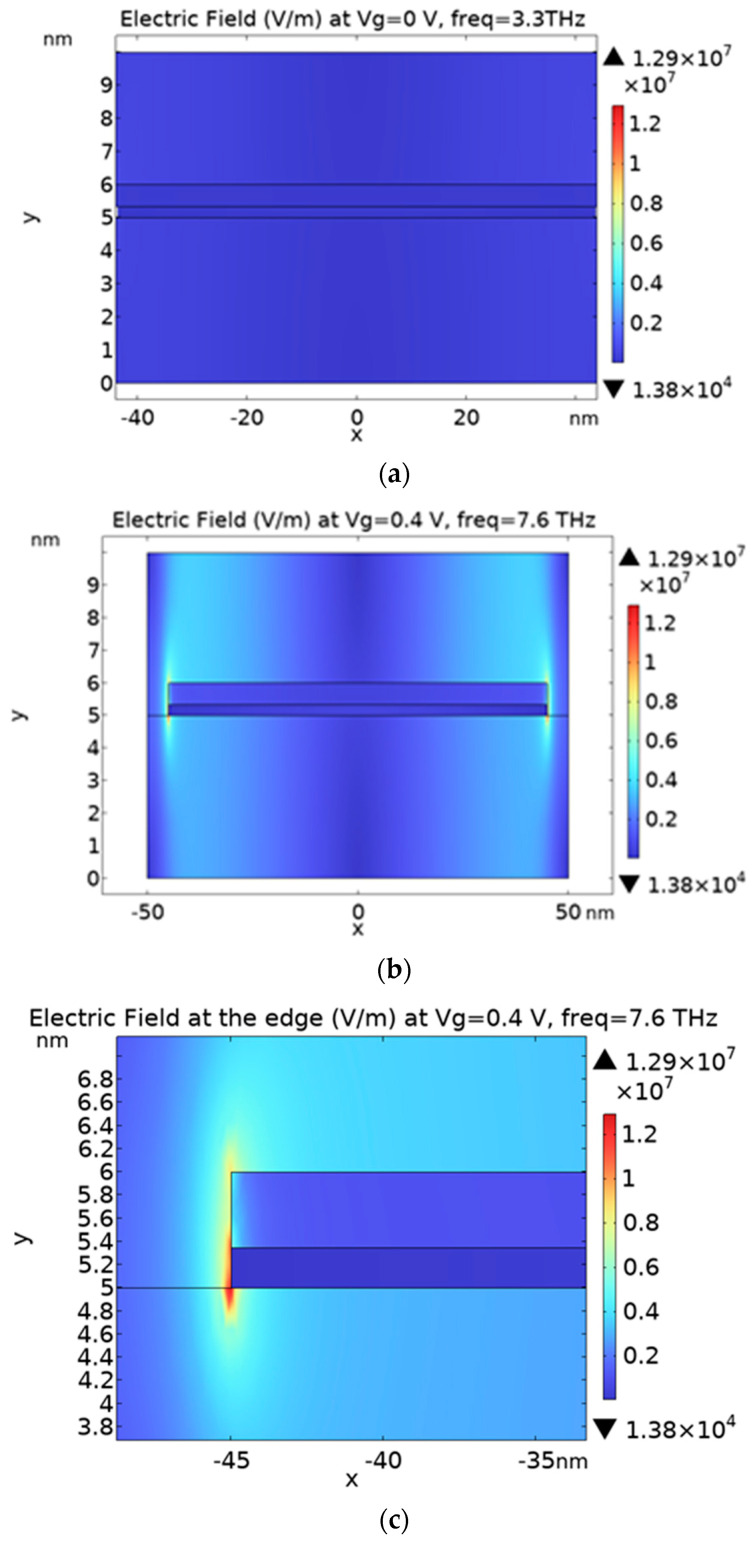
The electric field amplitude across one nanoribbon: (**a**,**b**) The electric field amplitude of a 90 nm width nanoribbon structure at $V_{g}=0$ and  0.4 V, respectively; (**c**) The zoom in of the nano-ribbon edges at $V_{g}=0.4\;V$.

Figure 4 shows the electric field distribution across the thickness of the nanoribbon heterostructure at the absorption frequency. The electric field is mostly concentrated at the edges of the nanoribbons and its magnitude increases when increasing the applied voltages. Graphene nanoribbon edges have higher electric field magnitudes than MoS_2_ due to its higher carrier concentration, which increases when increasing the applied voltage. It is to be noted that the electric field will be high at the corners regardless of the graphene orientation because the conductivity tensor for graphene is the same for both zigzag and armchair [42], and also, the edge effect will not be obvious for large finite size sheets (more than 125 nm width) [40,51].

### 3.2. The Effect of the Nanoribbon Width and Substrate Thickness

**Figure 5:**
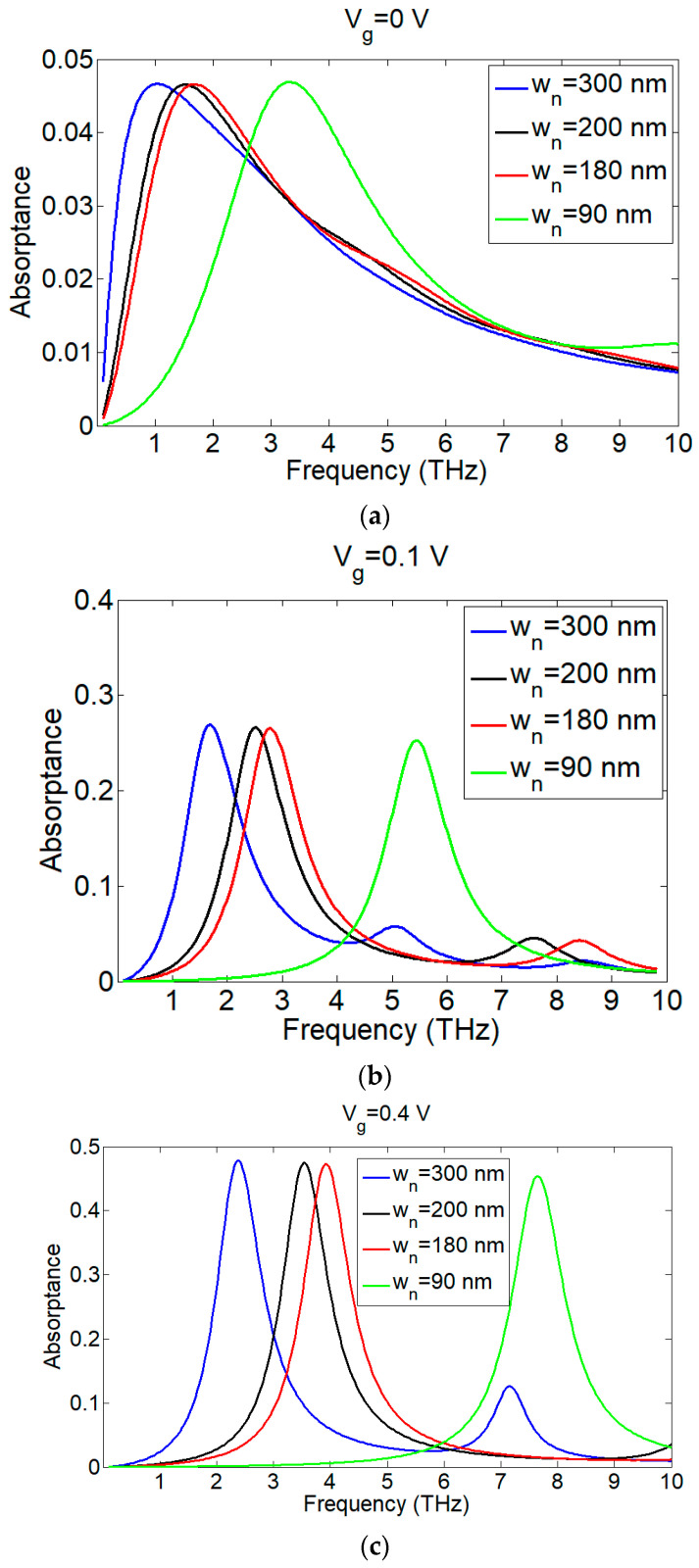
The effect of the nanoribbon width, $w_{n}$, on the absorptance frequency: (**a**) at V_g_ = 0 V; (**b**) at V_g_ = 0.1 V; (**c**) at V_g_ = 0.4 V.

**Figure 6:**
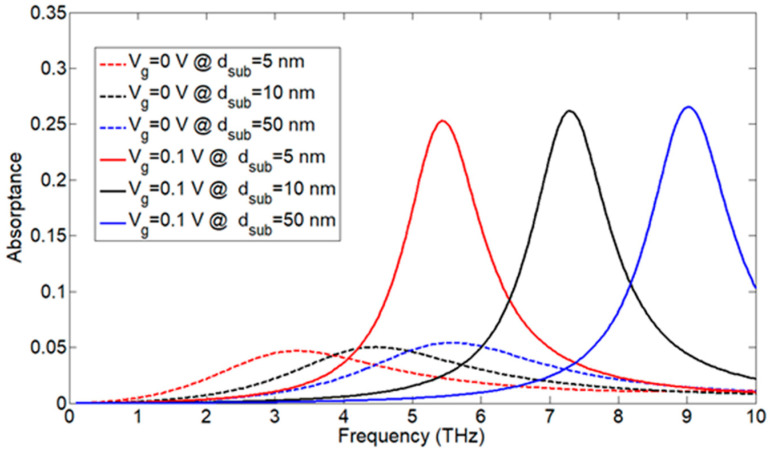
The effect of the substrate thickness, $d_{\mathrm{sub}}$, of SiO_2_ on the absorptance at Vg = 0, 0.1 V and nanoribbon width $w_{n}\;$ = 90 nm.

When sectioning graphene into nanoribbons, its bandgap opens up, and when decreasing the nanoribbon width, we obtain larger bandgaps [52]. Here, we varied the nanoribbon width, $w_{n}$, from 90 nm to 300 nm while keeping the same spacing, d , between the nanoribbons (10 nm). A shift in the absorption frequency towards low energy frequencies (low THz frequencies) occurred when increasing the nanoribbon width. Decreasing the widths to less than 90 nm blue-shifted the absorptance spectrum, outside the THz region in Figure 5c. Decreasing the nanoribbon width increased the bandgap; thus, we can have electron transitions at higher energies (frequencies). Increasing the substrate thickness of SiO_2_ ($d_{\mathrm{sub}})$ shifted the absorption frequency to higher frequencies and caused a little increase in absorption for different gate voltages, as shown in Figure 6. The blue shift in the absorption frequency with the substrate thickness is best described by the argument in [53], where a larger $d_{\mathrm{sub}}$ indicates a low capacitance value and low carrier concentration $n_{g}$ (Equation (6)), and since the resonance frequency or the absorption peak frequency follows the rule, $\omega \propto \frac{1}{\sqrt{LC}}$, where *L* and *C* are the equivalent inductance and capacitance of the structure, respectively; the resonance (absorption) frequency increases or shifts towards higher frequencies when the capacitance decreases. In order to have a good voltage tunable absorber for small nanoribbon widths (90 nm) in the THz region, we have to use a small substrate thickness in the range of 5 nm. This can be attributed to the fact that the carrier concentration of graphene is related to the substrate thickness according to Equation (6). We need a small $d_{\mathrm{sub}}$ (high equivalent capacitance) to have enough carrier concentration at the THz frequency.

### 3.3. The Effect of Temperature

**Figure 7:**
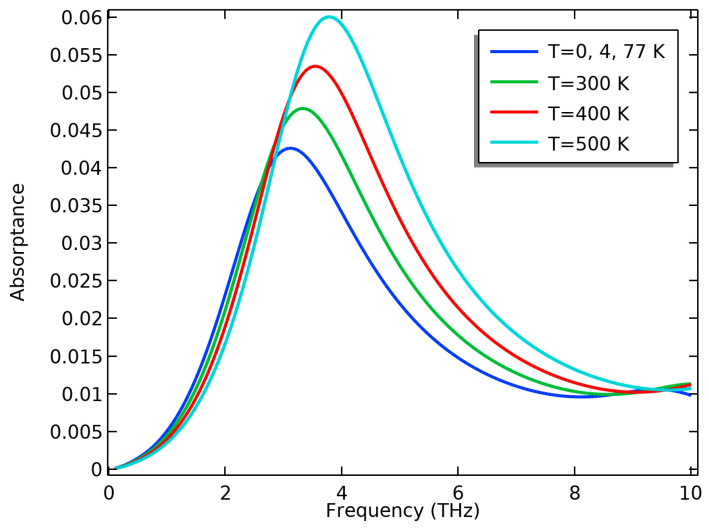
The effect of temperature on the absorptance of the nanoribbon structure at gate voltage V_g_ = 0.

**Figure 8:**
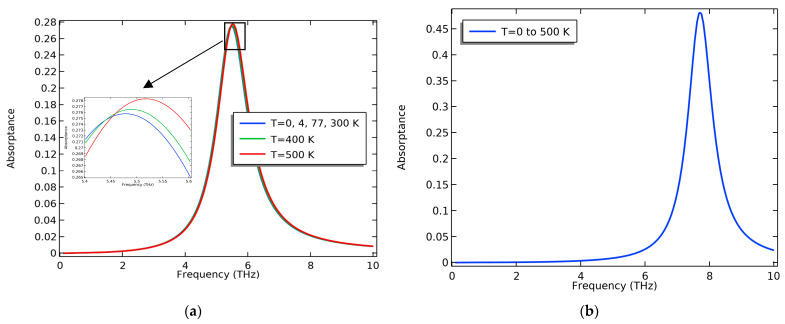
The effect of temperature on the absorptance of the nanoribbon structure at gate voltage (**a**) V_g_ = 0.1 V, and (**b**) V_g_ = 0.4 V.

Temperature can greatly affect the carrier concentrations in graphene nanoribbons; however, this effect will not be obvious for nanoribbon widths greater than 100 nm [26]. When increasing the temperature, the increase in carrier concentration was very low, especially in the presence of an applied voltage. At the same time, the transmittance of the MoS_2_ sheets slightly decreased with the increase in temperature [54]; that is why we could not see a great effect of temperature on the structure absorptance, especially at higher voltages. The experimental work in [55] showed that both the refractive index, n, and the extinction coefficient, κ**,** curves of MoS_2_ are not affected when varying the temperature from 4.5 K to 500 K as we go towards the THz region. The proposed nanoribbon structure is stable against temperature variations. Figure 7 shows a slight variation in absorptance above 300 K that vanishes at higher gate voltages, as in Figure 8.

## 4. Discussion

We have presented an ultrathin simple design for a monochromatic, narrow-bandwidth THz absorber with a thickness of approximately 7 nm. The absorptance frequency can be adjusted through the device dimensions (the nanoribbon width) or the applied voltage. When applying 0.4 V, we could achieve ~50% absorptivity at an absorptance frequency of 7.83 THz, a bandwidth of 0.8 THz, and a fractional bandwidth of $\frac{0.8}{7.83}=10.2\%$, which is a narrow bandwidth (Figure 8b). We could maintain the same absorptivity and bandwidth by varying the nanoribbon width (Figure 5c). On the other side, absorptance frequency tunability was achieved through the applied vertical voltage, but in this case, the absorptance decreased at lower voltages. It is to be noted that there are other absorbers that have achieved high absorptance from 70% to 99%, but they are intended for other applications, such as the multiband absorber in [56] where the maximum absorption was only achieved in a very small range of 0.1–2.0 THz; moreover, the structure is a complex metamaterial structure. The broadband absorbers in [57,58] had a broadband absorption from 2 to 6 THz and from 1 to 3 THz, respectively. However, varying the Fermi level of graphene affects the absorptivity and can decrease it below 40%. Still, these structures have a complicated design, and their dimensions are in the range of μm . Their high absorptance is limited to a certain band in the THz region. We summarize some of the previous work of THz absorbers in Table 1. In summary, the proposed work is a low-cost, easily fabricated, tunable narrowband absorber. Based on the graphene nanoribbon widths used, the structure is polarization-insensitive [42]. Future work could include modifying the structure for increased absorptivity while maintaining the simplicity of the structure.

## 5. Conclusions

Graphene nanoribbons have low absorption peaks in the THz range, where the absorption peaks can be slightly tuned through applying a gate voltage. MoS_2_ has high transmittance in the THz range and very low absorption governed by intraband transitions. Graphene-only or MoS_2_-only nanoribbons cannot achieve good absorptance and tunability in the THz range. Since a MoS_2_/graphene heterostructure has a wider bandgap than that of graphene and more carrier concentration than MoS_2_, it can afford better absorption. In this work, we studied the absorption of a large array of infinite MoS_2_/graphene nanoribbons on a SiO_2_ substrate in the THz range. The structure showed better absorption than that of graphene-only nanoribbons. The absorption frequency can be easily tuned through the whole THz range by varying the vertical gate voltage based on the optical conductivity tunability of graphene with its chemical potential. The absorption frequency is affected by the nanoribbon width and substrate thickness. The temperature has little effect on shifting the absorption frequency, so the structure performance is stable at different temperatures. The proposed structure represents a cheap, easily fabricated, and tunable THz frequency absorber that can be used in many applications such as sensing, cancer diagnosis [62], and communication.

## Figures and Tables

**Table 1:** List of previous THz absorbers.

Ref	Bandwidth	Absorptance	Structure
[56]	Multiband width	99.7% in a frequency range of 0.1–2.0 THz	Graphene meta surface, Au, SiO_2_, and Zeonex.
[57]	Relative bandwidth of 72.1% at 90% absorptance	>90% for frequencies from 2 to 5 THz	Gammadion-shaped graphene sheet and a polydimethylsiloxane dielectric substrate placed on a metal film.
[58]	Wide 1.2 to 2.67 THz	>90% for frequencies from 1.375 to 2.75 THz	Patterned MoS_2_ with a subwavelength ring–cross array.
[59]	~0.5 THz	>90% for frequencies from 4.762 to 5.152 THz	(Square ring absorber) of Au and Si_3_N_4_.
[60]	~5.8 THz	70% for frequencies from 9 THz to 7.7 THz	Ribbons of graphene on dielectric and Au film.
[61]	Ultrawideband 3.4–9.6 THz	>90% for frequencies from 4 to 10 THz	Frustum pyramid stack of BP/dielectric on a gold base.

## Data Availability

Not applicable.
